# Early Diagnosis of Problems Related to the Self-Organization of the Cardiovascular System Based on the Interplay between RR and JT Cardiac Intervals

**DOI:** 10.3390/diagnostics14131410

**Published:** 2024-07-02

**Authors:** Naseha Wafa Qammar, Minvydas Ragulskis, Loreta Saunoriene, Rasa Smidtaite, Alfonsas Vainoras, Gediminas Jaruševičius

**Affiliations:** 1 Department of Mathematical Modelling, Kaunas University of Technology, Studentu 50-147, LT-51368 Kaunas, Lithuania; 2 Institute of Cardiology, Lithuanian University of Health Sciences, A. Mickeviciaus g. 9, LT-44307 Kaunas, Lithuania; alfonsas.vainoras@lsmuni.lt (A.V.);

**Keywords:** cardiac interval, collapse of complexity, wavelet analysis

## Abstract

The dynamics of the collapse of complexity observable in the performance of the cardiovascular system during the stress test is investigated in this paper. For this purpose, the interplay between the RR and JT cardiac intervals is measured and assessed for each participant. This case study involves a modest sample size of eight individuals with normal and elevated blood pressure. Although it is anticipated that the interaction between the RR and JT intervals is rather complex during the stress test, the existence of interpretable time delays between those cardiac intervals is demonstrated using the time delayed patterns algorithm. The assessment of the cardiovascular mobilization taking place during the stress test is also an integral part of this study. The velocity of adaptation index $A_{d}$ and the newly formulated modified adaptation index $A_{r}$ (computed only for the recovery phase) are used to quantify the healthy mobilization of the cardiovascular system for each participant. The time frequency analysis of the difference signal between the RR and JT intervals is used to quantify the collapse of complexity around the load termination point. Finally, a semi-gauge indication tool is constructed to assess the overall goodness of the self-organization of the cardiovascular system during the stress test.

## 1. Introduction

Before delving into the literature review and introduction to this study, it is essential to establish the framework of this research while adhering to the primary objective of the study. The main idea behind the research is to propose a novel mathematical approach to demonstrate the interplay of the cardiac parameters, the RR and JT, which are crucial in quantifying the collapse of complexity and the self-organization of the cardiovascular system during the stress test. Also, this paper employs the idea of the adaptation index to evaluate the velocity of the cardiovascular mobilization during the recovery phase. Complex mathematical techniques and statistical analysis are an integral part of the study. First, the rationale for using the RR and JT cardiac intervals is presented and thoroughly discussed. The diagnostic and clinical significance of analyzing the relationship between those parameters is described in further detail. Following that, the idea of the velocity of adaptation is adopted for the description of the cardiovascular mobilization during the exercise. Finally, nonlinear time series analysis techniques are discussed and employed for the construction of quantifiable indicators capable to describe the performance of human cardiac vascular system during the exercise.

The manifestation of the interplay between the cardiac intervals uncovers the fundamental mechanisms of the cardiovascular system [1]. The significance of such an analysis lies in the early diagnosis of various cardiac illnesses, and therefore its importance cannot be ignored. However, the reciprocity between the cardiac parameters is complex, and thus there is no single perfect algorithm to examine the interaction between them [1,2]. As a result, the research arena is always at disposal for analyzing the dynamic relationship between the cardiac intervals by using various mathematical and computational approaches.

Every single beat of an ECG can be decomposed into cardiac intervals comprising the whole inter-beat interval (the RR interval), the P-wave (associated with atrial depolarization), the QRS-complex (associated with ventricular depolarization), the T-wave (associated with ventricular repolarization), and many other specific cardiac intervals such as QT and JT intervals [1,3,4,5]. The study on the dynamics of cardiac intervals has proven to be a powerful technique for the detection of abnormal cardiac rhythms such as atrial fibrillation [6], acute myocardial infarction [7], temporal lobe epilepsy [8] and other diseases [9].

In 1920, Bazzet [10] analyzed the relationship between RR and QT intervals which were later entitled the Bazzet’s correction index represented by the following formula: QTc=QTRR, where ${QT}_{c}$ is the corrected QT interval [11,12,13]. Bazzet’s work on the cardiac intervals is significant in standardizing the assessment of QT intervals across different heart rates, which has definite therapeutic significance [14]. The difference between the ${QT}_{c}$ and QRS yields ${JT}_{c}$ (where ${JT}_{c}$ is the corrected JT interval) [15]. However, in this work, we use the uncorrected JT interval (the difference between JT and QRS intervals). Despite many years of active research, the discussion continues whether the corrected ${JT}_{c}$ or the uncorrected JT is better suited for concrete applications [16]. An in-depth explanation for employing the uncorrected JT interval is discussed in [16]. The major reason for using the uncorrected JT interval is based on the fact that we are interested in algebraic relationships between different cardiac intervals (for example, JT, RR, QRS, etc.). Additional correction of individual cardiac intervals in respect of the RR interval (the inter-beat interval) only makes those relationships less representative [16].

In fact, the study in [17] has shown that the algebraic relationship between the RR and uncorrected JT cardiac intervals is capable of revealing subtle features of atrial blood pressure regulation during the stress test which are not observable otherwise [17]. Similarly, the study in [6] introduced a technique for the early detection of arrhythmic episodes based on algebraic relationships between the RR and the uncorrected JT and QRS cardiac intervals [6]. 

Another important part of this study is evaluation of the cardiovascular mobilization for each individual during the stress test. Cardiovascular mobilization during a physical exercise is defined as the velocity of adaptation of the cardiovascular system during the exercise [18]. Also, the velocity of adaptation aims to quantify the extent to which the body functions are mobilized during the execution of the exercise [18]. The adaptation index is mathematically presented as [18]

Ad=1n∑j=1n(JTjJTmax−RRjRRmax)

where ${RR}_{j}$ and ${JT}_{j}$ are cardiac intervals measured at the $j_{th}$ contraction of the heart; ${RR}_{max}$ and ${JT}_{max}$ denote the longest RR and JT cardiac intervals recorded during the exercise; n is the total number of heart contractions during the exercise. Regardless of the type of the physical exercise, the $A_{d}$ index provides important physiological information on the functionality of the cardiovascular system. Note that the observation window used to record RR and JT cardiac intervals includes both the load and the recovery phases. The recovery phase of the stress test plays a very important role in the self-organization of the cardiovascular system. It is well known that the initial few minutes of the recovery phase are especially important [19,20]. It is shown in [21] that the first 60 s of the recovery phase are associated to the parasympathetic reactivation. Moreover, ref. [22] claims that the reduced heart rate at the start of the recovery phase may be used as a forecast for the increased risk of mortality. Keeping the importance of the recovery phase in mind, we introduce the $A_{r}$ index which represents the adaptation of the cardiovascular system but during the recovery phase only. 

Over the years, state-of-the-art analysis methods, including both linear and nonlinear techniques, have been utilized to analyze the ECG signal for the diagnosis of various cardiac illnesses. The linear analysis techniques include (1) time domain analysis that employs the determination of statistical and geometric parameters of HRV [23], (2) frequency domain methods which include the spectrum analysis of the HRV [23], (3) the time-frequency domain method which uses the localization of spectral components of the HRV and proven to be an effective means of illustration and graphical visualization of the state of health of an individual’s cardiovascular system [23]. Similarly, the nonlinear ECG analysis techniques include detrended fluctuation analysis (DFA) [24], reconstructed phase space analysis [25], Lyapunov exponents [26,27], correlation dimension [28,29,30], recurrence plot and Poincaré plots [31]. Similarly, the perfect matrices of the Lagrange differences technique are used to find the algebraic relationships between the ECG parameters for determining the complexity of atrial blood pressure regulation during the stress test [17]. The study presented in [17] examines the cardiovascular response during the stress test which involves two distinct phases: the load phase and the recovery phase. In [17], the study placed particular emphasis on understanding the dynamics of the cardiovascular system during the load phase, specifically when the collapse of complexity occurs as the load is increased until reaching a specified limit according to the American Heart Association guidelines for exercise testing. The research in [17] demonstrates the capacity of the cardiovascular system to endure the load among the individuals (with normal ABP as well as with a history of hypertension) as the load is increased. While it was already anticipated that the two groups would exhibit distinct responses to the same exercise, some individuals from the normal blood pressure group showed cardiovascular responses similar to those with hypertension, indicating that physical fitness does not always correlate with cardiovascular resilience under load. The study in [17] adopted a unique matrix-based algorithm and a distinct phase space visualization technique that presents both quantitative and qualitative analyses of the individual cardiovascular system responses to elucidate the collapse of complexity during the load phase of the stress test for the entire cohort.

While the load phase gained a considerable attention in [17], the least focus is directed towards the recovery phase (the phase which starts as the load is terminated). A key hypothesis explored in this study refers to the self-organization of the cardiovascular system that occurs during the stress test. A deeper understanding of the recovery phase is also important. However, it is well known that the self-organization of the cardiovascular system may vary for healthy individuals as well as with the people who have a history of hypertension. There may be individuals from the group who may seem physically fit but their cardiovascular system response (self-organization during stress test) may not be responsive just as it was true for the load phase (collapse of complexity) as presented in [17]. In this regard, a distinct matrix-based algorithm as well as visualization techniques are adopted to understand the self-organization of the cardiovascular system during the stress test. Similarly, the PMLD algorithm is also used for the early detection of atrial fibrillation episodes as shown in [6]. The implementation of linear and nonlinear time series analysis techniques for the ECG signal is dependent upon the research question which needs to be addressed. As a result, both linear and non-linear approaches have been shown to be preferable in their ability to analyze the ECG signal. 

In this paper, ECG data are registered using the Kaunas Load system capable to synchronously identify different cardiac intervals for the cohort of eight individuals. The classical stress test is used to record the RR and JT cardiac intervals for each person. The objective of this study is multifold and can be summarized as follows: (1) It is well known that the algebraic relationship between the RR and JT cardiac intervals is dynamically varying in time and carries important information about the collapse of complexity during the load phase [16,17]. However, one of the objectives of this study is also to explore whether any time lag synchronization exists between those cardiac intervals during the stress test. For this purpose, the time-delayed pattern technique is proposed to identify and visualize the varying time delays between the RR and JT cardiac intervals. (2) It is also hypothesized that the difference signals computed for the RR and JT cardiac intervals carry a lot of important information for the identification of the collapse of complexity and the self-organization of the cardiovascular system during the stress test. (3) Though it is well established that the velocity of adaptation index computed during the load and the recovery phases plays an important role in determining the mobilization of the cardiovascular system, we move a step further and propose the concept of the modified velocity of adaptation which is computed only during the recovery phase. It is hypothesized that finding the subtle variations between the RR and JT cardiac intervals (particularly at the commencement of the recovery phase) provides important information about the cardiovascular mobilization. (4) The wavelet analysis approach is additionally employed to visualize the high frequency large amplitude oscillations when they appear in the difference signal between the RR and JT cardiac intervals. Finally, descriptive statistical analysis is employed to develop the indicators characterizing the health status of an individual’s cardiovascular system.

## 2. Materials and Methods

### 2.1. Ethical Statement

The current study met all the criteria for experimental ethics. The Kaunas Regional Ethics Committee for Biomedical investigations, No. BE-1-30, granted permission to conduct biomedical studies. The participants provided informed written consent to conduct the biomedical studies. 

### 2.2. Experiment Protocol

The experiment protocol was conducted for 8 participants. The protocol began on 4 June 2020. The experiment lasted around 60 min for one subject beginning at 09:00 h and ending at 18:05 h (for the entire cohort). Table 1 summarizes the experimental protocol.

### 2.3. Experiment Conditions

The RR and JT cardiac intervals are registered synchronously by using the Kaunas Load system during the stress test. The Kaunas Load system was developed at the Institute of Cardiology, Lithuanian University of Health Sciences. The stress test used in this study is the bicycle ergometry exercise. The initial load is set to 50 W and maintained steady for two minutes before being incrementally increased by 50 W. An individual must maintain a steady cycle spinning rate of 60 rpm during the whole exercise. The load phase of the stress test is terminated when the participant fails to sustain this spinning rate or when the first clinical symptoms for load limitation are recognized according to the American Heart Association (AHA). 

### 2.4. Participants

The group of individuals consists of eight physically active males who are not professional athletes. The average and standard deviation of age is 41.11±10.21 years; height 178.88±0.071; weight 80.53±10.01 kg; body mass index 25.10±2.06 kg/$m^{2}$. The eight-person cohort is divided into two groups: “healthy” persons, denoted as $H_{n}$ where H denotes the healthy cohort and the subscript n represents the person’s consecutive number. The same is true for “unhealthy” persons, who are denoted by $U_{n}$. It should be emphasized that the classification of healthy and unhealthy is based upon blood pressure anomalies among the candidates, as shown in [17].

### 2.5. The Algorithm for the Computation of Time-Delay Patterns 

Let us consider time series x and y. Let us define the set of indexes $I=\left\{k-R,k-R+1,\ldots ,k+R\right\}$, where R is the radius of averaging. Now, we let the time series y be shifted to the right by δ time-steps in respect to the time series x. Mean difference between the corresponding elements of $x_{k-R},x_{k-R+1},\ldots ,x_{k+R}$ and $y_{k+\delta -R},y_{k+\delta -R+1},\ldots ,y_{k+\delta +R}$ reads

$$D\left(k,\delta ,R\right)=\frac{1}{2R+1}\sum_{j\in I}\alpha_{j}\cdot \left(x_{j}-y_{j+\delta }\right),$$

where $\alpha_{j}$, j∈I are the weighting coefficients. 

**Figure 1:**
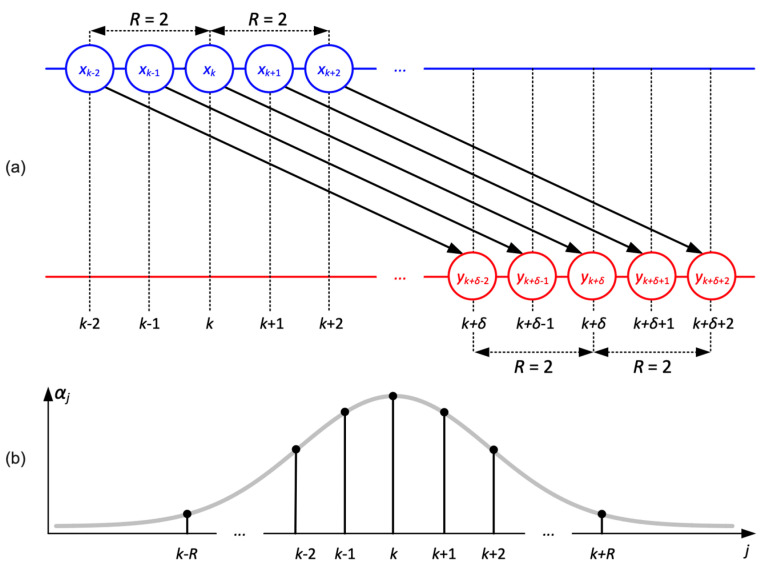
A schematic diagram illustrating the computation of time-delay patterns between time series x and y. Subplot (**a**) represents the schematic of the algorithm performed between the two-time series. Subplot (**b**) represents the differences that are weighted using a Gaussian distribution centered around k.

Numerical values of $\alpha_{j}(j\in I)$ should be preselected in such a way that the weights of the differences $x_{j}-y_{j+\delta }$ are distributed according to the Gaussian-type law centered around j=k. The values of $\alpha_{j}$ are determined using the continuous representation of the Gaussian-type distribution function: $f\left(t\right)=\frac{1}{\beta }e^{-\frac{1}{2}{\left(\frac{t-k}{\sigma }\right)}^{2}},$ where t is time; $\alpha_{j}=f\left(j\right),\;j\in I$; β is a constant such that $\sum_{j\in I}\alpha_{j}=1;$ and $\sigma^{2}$ defines the dominance of $\alpha_{k}$ with respect to all other weight coefficients as shown in Figure 1. Condition $\sum_{j\in I}\alpha_{j}=1$ holds true when $\beta =\sum_{l\in I}e^{-\frac{1}{2}{\left(\frac{l-k}{\sigma }\right)}^{2}}.$ The distribution of weight coefficients $\alpha_{j},\;j\in I$ becomes uniform when $\sigma \to \infty :\underset{\sigma \to \infty }{\lim}\alpha_{j}=\underset{\sigma \to \infty }{\lim}\frac{e^{-\frac{1}{2}{\left(\frac{j-k}{\sigma }\right)}^{2}}}{\sum_{l\in I}e^{-\frac{1}{2}{\left(\frac{l-k}{\sigma }\right)}^{2}}}=\frac{1}{2R+1}.$ On the other hand, the role of the surrounding differences vanishes when σ→0. This fact follows from the following identities: $\underset{\sigma \to 0}{lim}\;e^{-\frac{1}{2}{\left(\frac{s-k}{\sigma }\right)}^{2}}=0,\;s\in I\backslash ,\;e^{-\frac{1}{2}{\left(\frac{k-k}{\sigma }\right)}^{2}}=1.$ Then, β=1, $\underset{\sigma \to 0}{\lim}\alpha_{k}=1$, $\underset{\sigma \to 0}{\lim}\alpha_{s}=0\in I\backslash \{k\}$. We use R=2, σ=20 in all further computations. 

### 2.6. The Description of Panels A–F in the Figures

From the representation of the RR and JT cardiac intervals through preprocessing, the proposed mathematical algorithms, and wavelet analysis, all findings are depicted in a single figure with six panels for each person for the entire cohort. We discuss through each panel in detail in the sections that follow.

#### 2.6.1. Description of Panels A and B

##### Original and Denoised RR and JT Cardiac Intervals

**Figure 2:**
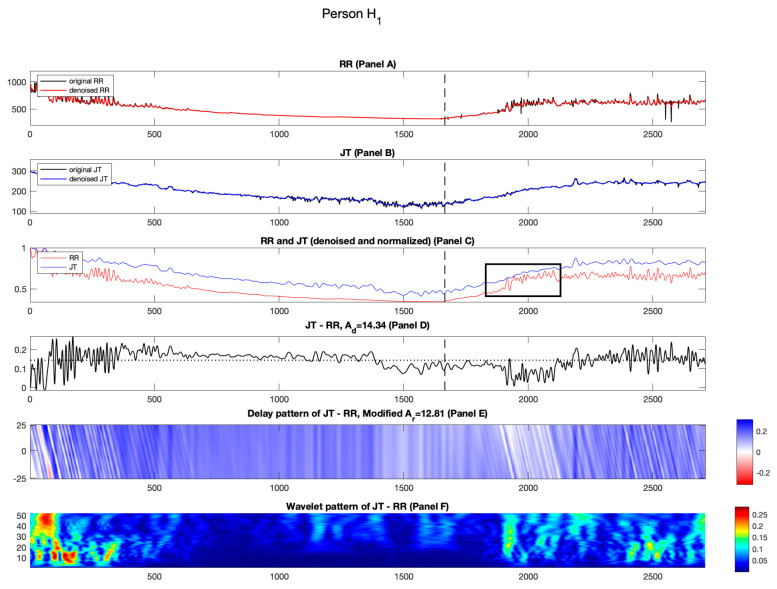
Illustration of the computational technique results for the healthy person $H_{1}$. Panel A: The original and denoised RR cardiac interval is shown in black and red. Panel B: The original and denoised JT cardiac interval is shown in black and blue. Panel C: The denoised and normalized RR and JT cardiac intervals are shown in red and blue. Panel D: The difference signal, JT−RR and the value of velocity of adaptation index $A_{d}$ is shown as a horizontal dashed line. Panel E: Delay pattern algorithm outcome is shown in a colormap pattern and the value of modified velocity of adaptation index $A_{r}$ computed during the recovery phase is also shown. Panel F: The outcomes of the Shan wavelet analysis performed on the difference signal is shown.

**Figure 3:**
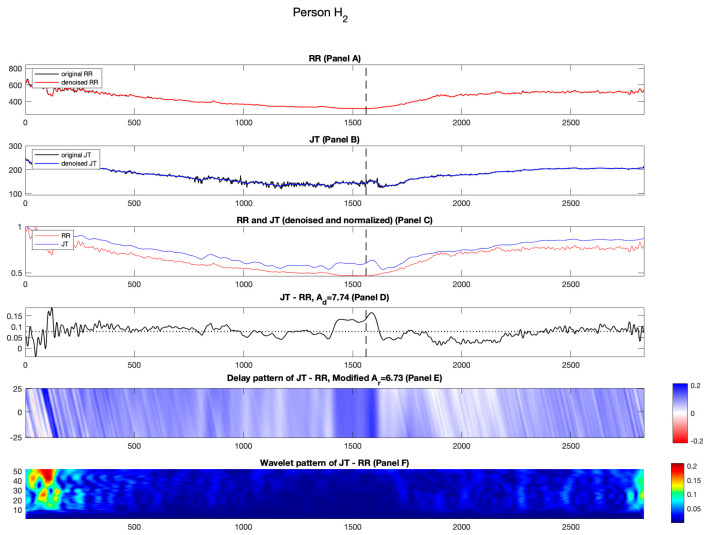
Illustration of the computational technique results for healthy person $H_{2}$. Panel A: The original and denoised RR cardiac interval is shown in black and red. Panel B: The original and denoised JT cardiac interval is shown in black and blue. Panel C: The denoised and normalized RR and JT cardiac intervals are shown in red and blue. Panel D: The difference signal JT−RR and the value of velocity of adaptation index $A_{d}$ are shown as a horizontal dashed line. Panel E: Delay pattern algorithm outcome is shown in a colormap pattern, and the value of modified velocity of adaptation index $A_{r}$ computed during the recovery phase is also shown. Panel F: The outcomes of the Shan wavelet analysis performed on the difference signal is shown.

**Figure 4:**
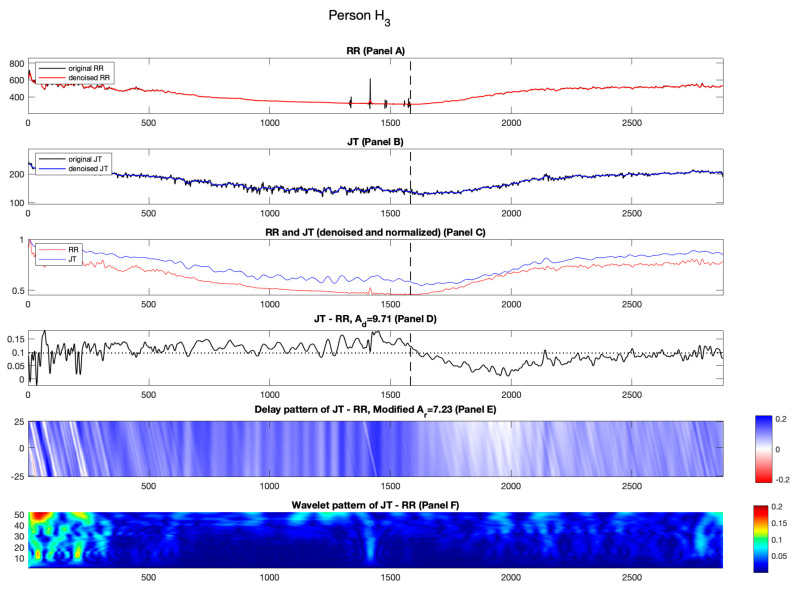
Illustration of the computational technique results for healthy person $H_{3}$. Panel A: The original and denoised RR cardiac interval is shown in black and red. Panel B: The original and denoised JT cardiac interval is shown in black and blue. Panel C: The denoised and normalized RR and JT cardiac intervals are shown in red and blue. Panel D: The difference signal JT−RR and the value of velocity of adaptation index $A_{d}$ are shown as a horizontal dashed line. Panel E: Delay pattern algorithm outcome is shown in a colormap pattern, and the value of modified velocity of adaptation index $A_{r}$ computed during the recovery phase is also shown. Panel F: The outcomes of the Shan wavelet analysis performed on the difference signal is shown.

**Figure 5:**
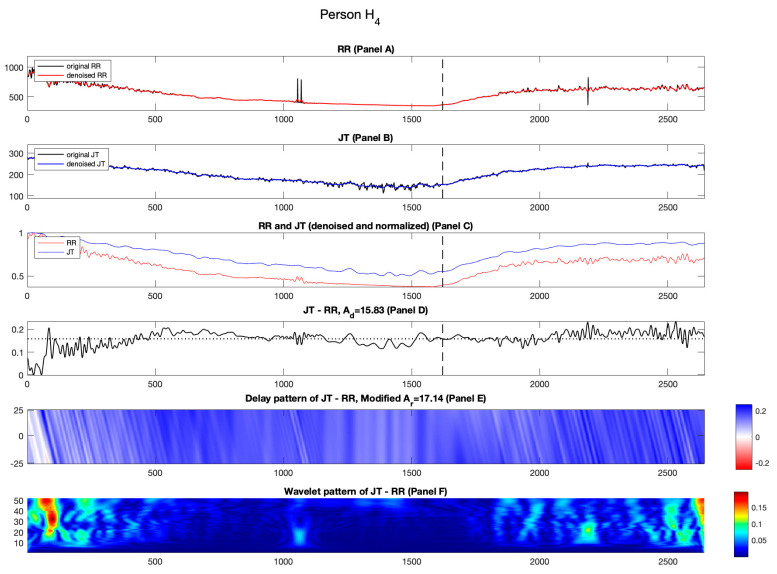
Illustration of the computational technique results for the healthy person $H_{4}$. Panel A: The original and denoised RR cardiac interval is shown in black and red. Panel B: The original and denoised JT cardiac interval is shown in black and blue. Panel C: The denoised and normalized RR and JT cardiac intervals are shown in red and blue. Panel D: The difference signal JT−RR and the value of velocity of adaptation index $A_{d}$ are shown as a horizontal dashed line. Panel E: Delay pattern algorithm outcome is shown in a colormap pattern and the value of modified velocity of adaptation index $A_{r}$ computed during the recovery phase is also shown. Panel F: The outcomes of the Shan wavelet analysis performed on the difference signal is shown.

**Figure 6:**
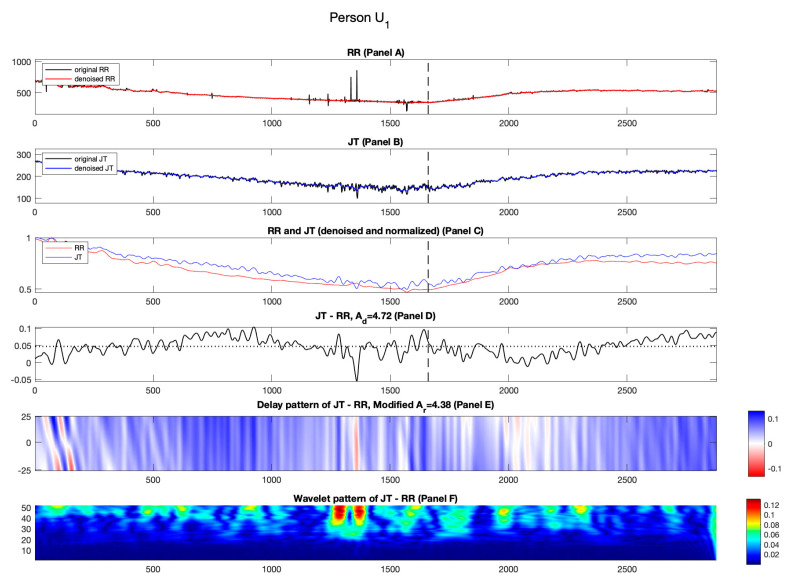
Illustration of the computational technique results for the unhealthy person $U_{1}$. Panel A: The original and denoised RR cardiac interval is shown in black and red. Panel B: The original and denoised JT cardiac interval is shown in black and blue. Panel C: The denoised and normalized RR and JT cardiac intervals are shown in red and blue. Panel D: The difference signal JT−RR and the value of velocity of adaptation index $A_{d}$ are shown as a horizontal dashed line. Panel E: Delay pattern algorithm outcome is shown in a colormap pattern and the value of modified velocity of adaptation index $A_{r}$ computed during the recovery phase is also shown. Panel F: The outcomes of the Shan wavelet analysis performed on the difference signal is shown.

**Figure 7:**
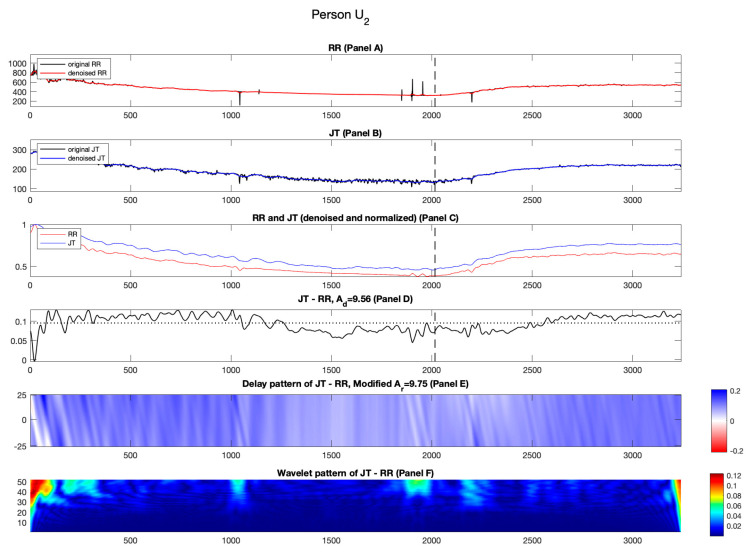
Illustration of the computational techniques results for unhealthy person $U_{2}$. Panel A: The original and denoised RR cardiac interval is shown in black and red. Panel B: The original and denoised JT cardiac interval is shown in black and blue. Panel C: The denoised and normalized RR and JT cardiac intervals are shown in red and blue. Panel D: The difference signal JT−RR and the value of velocity of adaptation index $A_{d}$ are shown as a horizontal dashed line. Panel E: Delay pattern algorithm outcome is shown in a colormap pattern and the value of modified velocity of adaptation index $A_{r}$ computed during the recovery phase is also shown. Panel F: The outcomes of the Shan wavelet analysis performed on the difference signal are shown.

**Figure 8:**
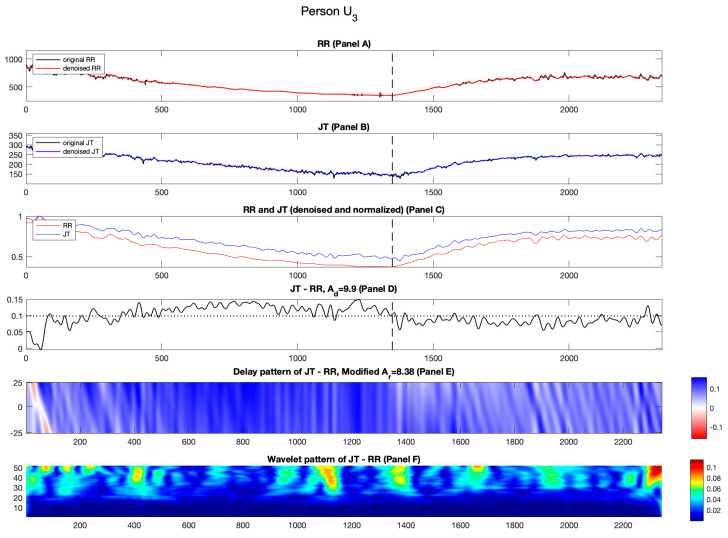
Illustration of the computational techniques results for unhealthy person $U_{3}$. Panel A: The original and denoised RR cardiac interval is shown in black and red. Panel B: The original and denoised JT cardiac interval is shown in black and blue. Panel C: The denoised and normalized RR and JT cardiac intervals are shown in red and blue. Panel D: The difference signal JT−RR and the value of velocity of adaptation index $A_{d}$ are shown as a horizontal dashed line. Panel E: Delay pattern algorithm outcome is shown in a colormap pattern and the value of modified velocity of adaptation index $A_{r}$ computed during the recovery phase is also shown. Panel F: The outcomes of the Shan wavelet analysis performed on the difference signal are shown.

**Figure 9:**
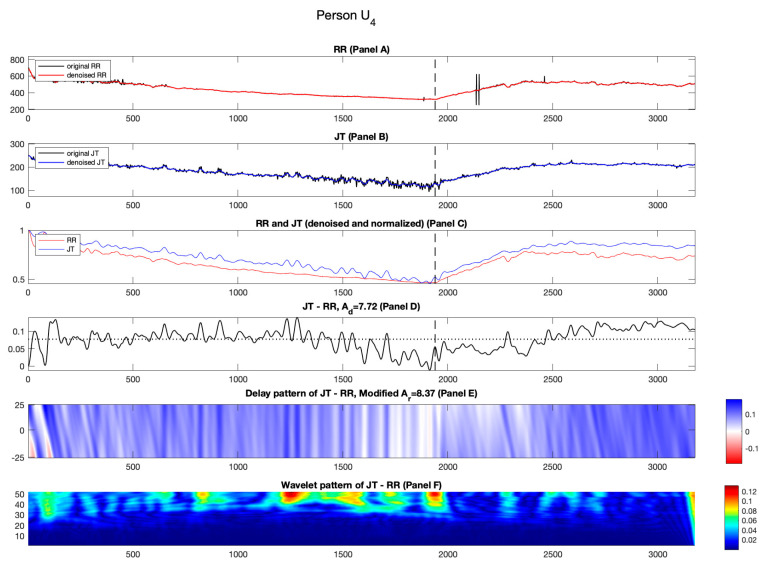
Illustration of the computational techniques results for unhealthy person $U_{4}$. Panel A: The original and denoised RR cardiac interval is shown in black and red. Panel B: The original and denoised JT cardiac interval is shown in black and blue. Panel C: The denoised and normalized RR and JT cardiac intervals are shown in red and blue. Panel D: The difference signal JT−RR and the value of velocity of adaptation index $A_{d}$ are shown as a horizontal dashed line. Panel E: Delay pattern algorithm outcome is shown in a colormap pattern and the value of modified velocity of adaptation index $A_{r}$ computed during the recovery phase is also shown. Panel F: The outcomes of the Shan wavelet analysis performed on the difference signal are shown.

The RR cardiac interval is shown in red in Figure 2, Figure 3, Figure 4, Figure 5, Figure 6, Figure 7, Figure 8 and Figure 9, Panels A. The x-axis represents the number of the cardiac cycle, whereas the y-axis represents the duration of the RR cardiac interval in milliseconds. As previously stated, the RR cardiac interval is recorded during the stress test, meaning that the load is consecutively increased by 50 W after each two minutes of the exercise until the load termination point. This load termination point is represented by a vertical dashed line. Denoising of the RR cardiac interval is accomplished using the variational mode decomposition approach as used in [32,33]. The denoised signal is shown in red in Figure 2, Figure 3, Figure 4, Figure 5, Figure 6, Figure 7, Figure 8 and Figure 9, Panels A. Similarly, the JT cardiac interval is represented in black in Figure 2, Figure 3, Figure 4, Figure 5, Figure 6, Figure 7, Figure 8 and Figure 9, Panels B. The x-axis represents the number of cardiac cycles, while the duration of the JT cardiac interval is shown on the y-axis in milliseconds. A vertical dashed line again indicates the load termination point. Denoising the JT series is achieved in the same way as for the RR cardiac interval. The denoised signal is presented in blue in Figure 2, Figure 3, Figure 4, Figure 5, Figure 6, Figure 7, Figure 8 and Figure 9, Panels B.

#### 2.6.2. Description of Panel C

##### Normalization

**Figure 10:**
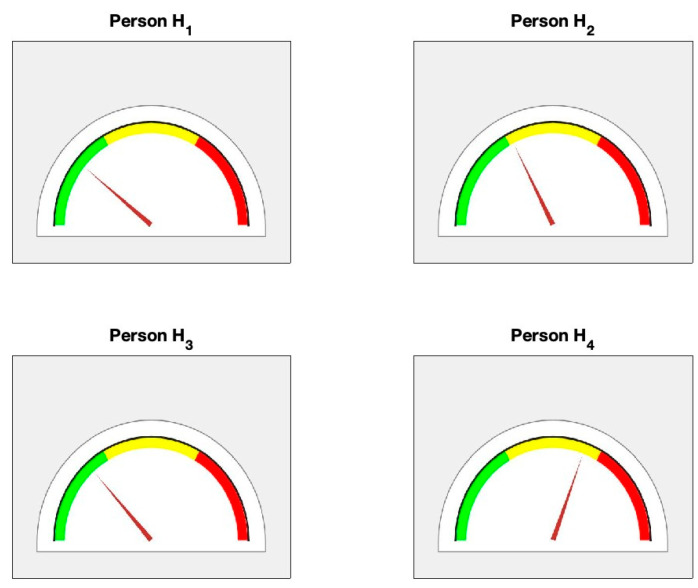
Illustration of the overall health status for the cohort of healthy persons ($H_{1}-H_{4}$) using the semi-gauge indication tool.

Both the RR and JT cardiac intervals are normalized according to formula xkxmax and ykymax, where $x_{k}$ and $y_{k}$ stands for the RR and JT cardiac intervals, respectively; $x_{max}$ and $y_{max}$ stand for the maximal duration of the RR and JT cardiac intervals, respectively, and (k=1,2,3,….) applies during the whole period of registration. In Figure 2, Figure 3, Figure 4, Figure 5, Figure 6, Figure 7, Figure 8 and Figure 9, Panels C, the x-axis denotes the number of cardiac cycles. The y-axis displays the amplitude of normalized RR and JT cardiac intervals. Both the normalized and denoised RR and JT cardiac intervals are represented in red and blue in Figure 2, Figure 3, Figure 4, Figure 5, Figure 6, Figure 7, Figure 8, Figure 9 and Figure 10, Panels C. The load termination is also depicted in Panels C by a vertical black dashed line.

#### 2.6.3. Description of Panel D

##### The Difference Signal and the Velocity of Adaptation Index, $A_{d}$

The difference signal is computed by subtracting the RR and JT cardiac intervals and is shown in Figure 2, Figure 3, Figure 4, Figure 5, Figure 6, Figure 7, Figure 8 and Figure 9, Panel D. The x-axis represents the number of cardiac cycles, while the y-axis represents the amplitude of the difference signal. Then, the velocity of the adaptation index is computed using formula Ad=1n∑i=1n(JTiJTmax−RRiRRmax)×100 as proposed in [18]. The value of $A_{d}$ is shown as a black dashed horizontal line across Panel D, on top of the difference signal. As before, the vertical dashed line indicates the load termination point.

#### 2.6.4. Description of Panel E

The algorithm for the computation of time-delay patterns is well explained in the previous section (see Figure 1). The outcomes of the algorithm are visualized in Figure 2, Figure 3, Figure 4, Figure 5, Figure 6, Figure 7, Figure 8 and Figure 9 in Panel E for each individual for the entire cohort.

##### The Modified Adaptation Index during the Recovery Phase, $A_{r}$

As mentioned previously in the introduction section, the initial few minutes of the recovery phase are also crucial in providing important information about the self-organization of the cardiovascular system [19,20]. Therefore, an in-depth analysis of the recovery phase is performed by averaging the time-shifted difference between the RR and JT cardiac intervals for the entire cohort. Thus, instead of averaging the scalar difference signal, we evaluate (average) the whole delay pattern after the termination of load. The numerical value of this averaging is denoted as the modified adaptation index during the recovery phase (see Figure 2, Figure 3, Figure 4, Figure 5, Figure 6, Figure 7, Figure 8 and Figure 9, Panel E). 

#### 2.6.5. Description of Panel F

The complex continuous wavelet analysis (Shan wavelet) is performed upon the difference [34]. The wavelet analysis helps to analyze the high-frequency large-amplitude oscillations occurring during the stress test for each person. These oscillations carry the useful information about the cardiovascular collapse of complexity during the exercise. To conduct the wavelet analysis, the built-in Matlab analysis feature is used. A few of the basic features of the wavelet analysis include the color map, number of colors, the coloration mode, the sampling period, etc., and an explanation of these features is provided as follows: (1) Coloration is performed with taking into account all coefficient values of the difference signal; (2) the wavelet coefficient at all scales is used to scale the coloration; (3) the sampling period for the analysis is set to the default, which is one second; and (4) jet colors are used to better visualize the coloration variations; see Figure 2, Figure 3, Figure 4, Figure 5, Figure 6, Figure 7, Figure 8 and Figure 9, Panel F.

### 2.7. The Statistical Analysis 

The statistical analysis in this study contributes to the development of two health indicators (named α and β) used to assess the health status of an individual’s cardiovascular system. Each of the health indicators is built on the information retrieved from specialized statistical analysis of the RR and JT cardiac intervals recorded for the whole cohort (including healthy and unhealthy individuals). Furthermore, this study provides a qualitative and quantitative foundation for the development of an open architecture for the assessment of the response of the cardiovascular system during the stress test. First, the proposed algorithm can accommodate a larger number of health indicators. The proposed health indicators α and β are only used as typical indicators in this study. Second, the number of individuals could be seriously enlarged. This case study includes only eight individuals, which, although it a very small sample size, is sufficient to demonstrate the capabilities of the proposed algorithms and statistical techniques. Similar approaches are also presented in [6,17], where health indicators are utilized to classify individuals based on statistical techniques.

This paper demonstrates that subtle relationships between cardiac intervals can serve as a powerful tool for designing novel computational techniques capable to perform sensitive detection of small changes in the self-organization of individual’s cardiovascular system. The statistical analysis of the study is carried out in two steps, which are described below in detail.

#### 2.7.1. Step A

In the first step, the adaptation index $A_{d}$ and the modified adaptation index $A_{r}$ are processed according to formula $X=\frac{\left(A_{d}-A_{r}\right)}{A_{d}}\times 100\%,$ where X is computed for each of the eight individuals in the entire cohort. The subtraction of the $A_{d}-A_{r}$ is in line with the fact that after the load termination, the self-organization of the cardiovascular system takes place. At the beginning of the recovery phase, the RR cardiac interval tries to return back to normal faster than the JT cardiac interval [19,20]. This effect is illustrated in the black box in Figure 2, Panel C. Also, this effect is represented by the white streaks in Panel E in the delayed pattern. We note that this effect is characteristic of healthy individuals only and automatically results in the lower values of the $A_{r}$ index compared to the $A_{d}$ index. The values of X computed for the entire cohort result in the minimum and maximum of X which are chosen to produce the health indicator α which is given as $\alpha =\frac{X-minX}{maxX-minX}\times 100\%$ (see Table 2), where minX is the minimum value of X and maxX is the maximum value of X in the whole cohort (including healthy and unhealthy individuals). 

#### 2.7.2. Step B

The high-frequency large-amplitude oscillations of the JT−RR signal can be observed at the beginning of the exercise and at the end of the recovery phase (Figure 2, Panel D). However, the variability of this signal diminishes considerably in the region of the high load which is represented by yet another indicator, β. Thus, we employ the wavelet analysis to the JT−RR signal to visualize the mentioned effect of the loss of variability in the region of the high load (this effect is again observable for healthy individuals). The following sub-steps are executed to compute the values of indicator β:

(1) Shan wavelet analysis is performed for the JT−RR signal. The resulting pattern of Shan wavelet coefficients is divided into three segments in such a way that the first segment represents the first 900 data values, the second section represents the next 900 data values, and the last section comprises all the remaining data points. Such a division of the pattern into three segments guarantees that the moment of the load termination falls into the second segment (note that this moment is different for each individual and depends on many factors characterizing the physical performance of that individual).
(2) The mean of each of the three segments is computed for each individual (for the entire cohort including healthy and unhealthy individuals) and labelled as $C_{1}$, $C_{2}$, and $C_{3}$ (see Table 3).
(3) A quadratic equation is interpolated through three points in a plane: $\left(-1,C_{1}\right)$, $\left(0,C_{2}\right)$, $\left(1,C_{3}\right)$. The equation of the parabola reads ${ax}^{2}+bx+c$. We are interested only in the parameter a because it defines whether the branches of the parabola move up or down. In other words, we are interested in whether the values of the Shan wavelet coefficients are smaller in the middle segment of the pattern. In this way, parameter a becomes a significant indicator distinguishing the healthy and unhealthy persons within the cohort. The computation of the value of parameter a is straightforward: $a=(C_{1}+C_{3})/2-C_{2}$.
(4) Step 3 yields parameter a for each individual. Repeating the process for the whole cohort yields the minimum and maximum values for the entire cohort (see Table 3).
(5) Indicator β is defined as follows: $\beta =\frac{a-mina}{maxa-mina}$, where mina is the minimum value of a and maxa is the maximum value of a for the whole cohort (including healthy and unhealthy individuals).
(6) Finally, the parameter representing the health status of an individual’s cardiovascular system is computed as γ=(α+β)/2 (see Table 4).

**Table 3:** Formulization of the health indicator β for each individual for the entire cohort.

Mean of Wavelet for First Slot, $C_{1}$	Mean of Wavelet for Second Slot, $C_{2}$	Mean of Wavelet for Last Time Slot, $C_{3}$	Equation of Parabola, $y={ax}^{2}+bx+c,$ $C_{n}=\left\{\begin{array}{ccc} a-b+c, & x=-1, & n=1\\ c, & x=0, & n=2\\ a+b+c, & x=1, & n=3 \end{array}\right.$ $a=(C_{1}+C_{3})/2-C_{2}$	Parameter, $\beta =\frac{a-mina}{maxa-mina}$
0.0536	0.0252	0.0587	3.09 *	100
0.0308	0.0063	0.0183	1.82	66.8
0.0322	0.0159	0.0203	1.03	46.2
0.0302	0.0103	0.0346	2.21	77.02
0.0174	0.0253	0.0183	−0.74 **	0
0.0139	0.0056	0.0100	0.63	35.7
0.0188	0.0206	0.0217	−0.03	18.5
0.0192	0.0215	0.0144	−0.47	7.04

The maximum value is indicated by an asteria * whereas the minimum value is indicated by double asteria **.

**Table 4:** Evaluation of the health status of an individual cardiovascular system.

α	β	Evaluation of the Health Status of an Individual Cardiovascular System, γ=(α+β)/2
56.17	100	78.08
63.18	66.8	64.99
100	46.2	73.1
0.41	77.02	38.71
45.97	0	22.98
18.93	35.7	27.31
69.98	18.5	44.24
0	7.04	3.52

## 3. Results and Discussion

### 3.1. Healthy Cohort

Figure 2, Figure 3, Figure 4 and Figure 5 show the results for healthy individuals $H_{1}-H_{4}$. For healthy person $H_{1},$ the original and denoised RR and JT cardiac intervals are shown in red and blue in Panels A and B, Figure 2. The load and the recovery phases of the stress test are divided by a vertical dashed line (which corresponds to the load termination point). For individual $H_{1}$, the load termination point or the highest load an individual can endure while performing bicycle ergometry test is 325 W. In Panel C, the RR and JT cardiac intervals are normalized using the algorithm described in the Methods section. In Panel C, the x-axis shows the number of cardiac cycles and the y-axis represents the amplitude of the normalized signals. As hypothesized and mentioned in [19,20], the initial minutes of the recovery phase are important in providing the information about the self-organization of the cardiovascular system. This effect is highlighted by a black box in Figure 2, Panel C. It can be observed that the normalized RR cardiac interval almost reaches the normalized JT cardiac interval during the second minute of the recovery phase. However, such intermittent increase in the normalized RR cardiac interval appears to happen too soon. In other words, the cardiovascular system tries to lower the heartbeat rate (without according lowering of the normalized JT cardiac interval). It appears that such short-term autoregulation does not last long. The general recovery process lasts much longer (Figure 2 Panel C). Similar effects are also observable for healthy persons $H_{2}$ and $H_{3}$ (see Figure 3 and Figure 4, Panel C). 

As discussed in the Methods section, we wish to explore not only the relative distances between the normalized RR and JT cardiac intervals but also the effects induced by time lags between those intervals. Panel E in Figure 2 highlights the importance of the initial few minutes of recovery. Figure 2, Panel E shows the delayed pattern between the normalized JT and RR cardiac intervals. The effects discussed in the previous paragraph are represented by white streaks in Figure 2, Panel E during the second minute of the recovery phase. The average of the delayed pattern after the termination of the load yields parameter $A_{r}$. It is natural to expect that the numerical value of $A_{d}$ is lower than $A_{d}$ for person H_1_ ($A_{r}=12.01\%$; $A_{d}=14.34\%$). The same effects are also observable for healthy persons $H_{2}$ and $H_{3}$ (Figure 3 and Figure 4, Panel D; also, see Table 2). 

Panel D in Figure 2 depicts the difference signal between normalized JT and RR cardiac intervals. It is well known that the effect of the collapse of complexity happens before the termination of the load [16]. Panel D in Figure 2 is yet another representation of the collapse of complexity. The variability of the difference signal is relatively high at the beginning of the stress test (Figure 2, Panel D). The variability of the difference signal then drops significantly before the termination of the load and keeps low at the beginning of the recovery phase. Later, the variability of the difference signal grows again.

The described effect of the collapse of complexity is well represented by the pattern of Shan wavelet coefficients in Figure 2, Panel F. The high variability of the difference signal is represented by bright regions in the pattern of the Shan wavelet coefficients. As mentioned in the Methods section, Shan wavelet analysis is performed for the difference signal between normalized JT and RR intervals. The resulting pattern of Shan wavelet coefficients is divided into three segments. For the individual $H_{1},$ the values of the means computed in each of the three segments are $C_{1}=0.0536;\;C_{2}=0.0252;\;C_{3}=0.0587;$ accordingly. As the load termination point occurs in the second segment, it is observable that the mean value of the Shan wavelet pattern in the second segment is lower than in the other two segments. This feature is observed for the healthy individuals (see Table 3). 

To represent the effect of the collapse of complexity during the stress test, a second-order polynomial is interpolated through the following three points in a plane: $\left(-1,C_{1}\right)$, $\left(0,C_{2}\right)$ and $\left(1,C_{3}\right)$. The algebraic equation of the parabola interpolating these three points reads *y* = ${ax}^{2}+bx+c$. As mentioned previously, we are only interested in parameter a because it defines whether the branches of the parabola move up or down. Eventually, parameter α serves as a signature for quantifying the collapse of complexity. For the individual $H_{1}$, the value of a is 3.09 (see Table 3). It can also be observed that the value of parameter a is always greater than zero for the healthy cohort $H_{1}-H_{4}$ (see Table 3). 

Interesting results are seen for healthy individual $H_{4}$. In Figure 5, Panel C, it is observed that the normalized RR cardiac interval almost drifts away from the normalized JT cardiac interval and stays away until the end of the recovery phase (Figure 5, Panel C).

Figure 5, Panel E also highlights the importance of the initial few minutes of the recovery phase by analyzing the effects induced by time lags between the normalized RR and JT cardiac intervals. This effect is seen by the presence of blue streaks in Figure 5, Panel E (unlike the other healthy individuals where this effect is observed with white streaks). This results in greater numerical value of $A_{r}$ compared to $A_{d}$. For person $H_{4}$, ($A_{r}=17.14\%$; $A_{d}=15.83\%$). 

Panel D in Figure 5 is another representation of the collapse of complexity. Of course, the variability of the difference signal is relatively high at the beginning of the stress test (Figure 5, Panel D). Later, the variability of the difference signal drops significantly and stays low until the end of the recovery phase (see Panel D, Figure 5).

Another representation of the collapse of complexity is shown by the pattern of Shan wavelet coefficients in Figure 5, Panel F. Like the other healthy individuals, the high variability of the difference signal is represented by bright regions in the pattern of the Shan wavelet coefficients for $H_{4}$. The values of the mean computed in each of the three segments are $C_{1}=0.0302;\;C_{2}=0.0103;\;C_{3}=0.0346$. Similar to the behavior of the other healthy individuals, it is observable that the mean value of the Shan wavelet pattern in the second region is lesser than the other two regions. Also, the value of parameter a is 2.21, which is greater than zero (an attribute noticeable for the healthy persons only). 

The quantifiable measure of the collapse of complexity appears to be similar for all the healthy individuals in the cohort (see Table 3). However, when the recovery phase alone is analyzed for the entire healthy cohort, it is observed that the self-organization of healthy individual $H_{4}$ exhibits a different behavior compared to other individuals from the healthy cohort $H_{1}-H_{3}$ (see Figure 5).

### 3.2. Unhealthy Cohort

#### 3.2.1. Candidate U_1_

Figure 6, Figure 7, Figure 8 and Figure 9 depict the results for unhealthy individuals $U_{1}-U_{4}$. When saying “unhealthy”, we mean that the individuals have a diagnosed elevated blood pressure. The maximum load the individual $U_{1}$ can bear during the exercise is 325 W which is indicated by a vertical dashed line in Figure 6, Panels A and B. Specific effects of the self-organization of the cardiovascular system can be observed during the recovery phase for person $U_{1}$ in Figure 6, Panel C. The normalized RR cardiac interval almost reaches the normalized JT cardiac interval right after the termination of the load and stays there for the first two minutes of the recovery phase. In general, the recovery after the physical exercise takes a loner time compared to healthy persons (see Figure 6, Panel C). 

The effects induced by the time lags between normalized RR and JT cardiac intervals are observed during the recovery phase in Panel E, Figure 6. These effects are represented by a mix of red and white streaks in Figure 6, Panel E. The red and white streaks continue as the recovery begins and stays during the first initial minutes of the recovery phase. This effect leads to a greater value of $A_{d}$ compared to $A_{r}\;$($A_{d}=4.72\%$; $A_{r}=4.38$%). The greater values of $A_{d}$ are also observed for the cohort of healthy persons $H_{1}-H_{3}$ (see Table 2).

Panel D in Figure 6 is another representation of the collapse of complexity. Though the variability of the difference signal is relatively high at the beginning of the stress test, the streaks of red colors remain observable until the load termination point and at the beginning of the recovery phase (Panel D, Figure 6). 

Figure 6, Panel F shows the pattern of Shan wavelet coefficients which serves as another representation of the collapse of complexity. The variability of the difference signal is high at the beginning of the load and stays high until the load termination point (and even a few minutes at the beginning of the recovery phase). The mean values of the three segments of the wavelet coefficients are ($C_{1}=0.0174;\;C_{2}=0.0253;\;C_{3}=0.0183$). Note that the middle segment produces the larger mean value as compared to the other segments. This quantifiable behavior is totally the opposite to what we have observed for the healthy cohort. This leads to a negative value of parameter a (−0.74) which is a noticeable signature of the unhealthy cohort (see Table 3).

#### 3.2.2. Candidate U_2_

Figure 7 shows the outcomes for unhealthy person $U_{2}$. The normalized RR cardiac interval drifts away from the normalized JT cardiac interval during the recovery phase (Figure 7, Panel C). 

The effects induced by the time lags between normalized RR and JT cardiac intervals are represented by streaks of blue and white spread across the axis (Figure 7, Panel E). These effects generate a higher value of $A_{d}$ compared to $A_{r}\;$($A_{d}=9.56\%$; $A_{r}=9.75$%). Note that larger values of $A_{d}$ are also a common signature for the cohort of healthy persons (see Table 2). The variability of the difference signal remains smooth throughout the stress test (unlike other individuals in the unhealthy cohort) (Figure 7 Panel D). 

Figure 7, Panel F shows the pattern of Shan wavelet coefficients. The variability of the difference signal is rather smooth throughout the stress test and stays same until the end of the recovery phase. The mean values of the three segments of the wavelet coefficients read $C_{1}=0.0139;\;C_{2}=0.0056;\;C_{3}=0.0100$. The middle segment produces the lower mean value compared to the other segments. This effect produces a smaller value of parameter a (0.63) (see Table 3). 

#### 3.2.3. Candidate U_3_

The normalized RR and JT cardiac intervals do not come closer to each other for unhealthy person $U_{3}$ during the recovery phase (Figure 8, Panel C). 

The streaks of blue and white spread across the load and the recovery phase (Figure 8 Panel E) which results in the higher value of $A_{d}$ compared to $A_{r}\;$($A_{d}=9.9\%$; $A_{r}=8.38$%). Such greater values of $A_{d}$ are also observed for the cohort of healthy persons $H_{1}-H_{3}$.

The variability of the difference signal remains mostly unchanged throughout the stress test (Figure 8, Panel D). The mean values of the three segments of the wavelet coefficients read $C_{1}=0.0188;\;C_{2}=0.0206;\;C_{3}=0.0217$. The resulting value of parameter a is −0.03 (Table 3).

#### 3.2.4. Candidate U_4_

Figure 9, Panel C shows the results for unhealthy person $U_{4}$. The normalized RR cardiac interval comes closer to the normalized JT cardiac interval at the beginning of the recovery phase, and then later moves away. 

The effects induced by the time lags between normalized RR and JT cardiac intervals can be observed during the recovery phase (Figure 9, Panel E). These effects are represented by streaks of blue and white. This effect generates lower value of $A_{d}$ compared to $A_{r}\;$($A_{d}=7.72\%$; $A_{r}=8.37$%), an attribute also observed for unhealthy persons $U_{1}$ and $U_{2}$. 

It can be observed that the variability of the difference signal is high throughout the load and the recovery phases (Figure 8 Panel F). The mean values of the three segments of the wavelet coefficients are ($C_{1}=0.0192;\;C_{2}=0.0215;\;C_{3}=0.0144$). The middle segment produces the higher mean value compared to the other two segments. This again results in a negative value of parameter a (−0.47) (see Table 3).

Finally, parameter α is computed using the values of $A_{d}$ and $A_{r}$ for the entire cohort (see Table 2). Also, parameter β is calculated using the mean values from the Shan wavelet analysis (Table 3). In the next step, values of ∝ and β are normalized between 0 and 100% (Table 2 and Table 3).

**Figure 11:**
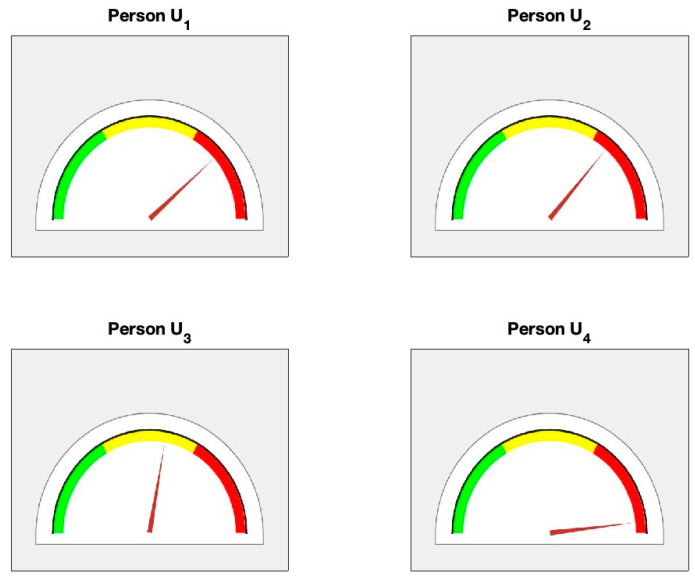
Illustration of the overall health status for the cohort of unhealthy persons ($U_{1}-U_{4}$) using the semi-gauge indication tool.

Based upon the values of α and β, indicator γ is calculated as the arithmetic average of α and β accordingly (Table 4). Finally, a semi-gauge health indication tool is produced (see Figure 10 and Table 4). Individuals $H_{1}$, $H_{2}$, $H_{3}$ are in the green zone in terms of cardiovascular health, but there is a caution for individual $H_{4}$ since it is in the yellow zone in the semi-gauge indication tool (because recovery indicator α did not produce such a large value as expected for a healthy person). It should be noted that individuals in the healthy cohort are all classified as healthy individuals based on blood pressure regulation; however, with this proposed technique, individual $H_{4}$ is ruled out from healthy cohort even though they have normal blood pressure. However, given their recovery dynamics, individual $H_{4}$ remains in the warning zone. For unhealthy cohort $U_{1}$, $U_{2}$ and $U_{4}$ are classified as unhealthy individuals; however, individual $U_{3}$ stays in the yellow zone (their recovery phase looks excellent) see Figure 11.

During physical exercise, the individuals with blood pressure anomalies may undergo hypertensive responses and enter a hypertonic state [17]. The exploration of the self-organization of the cardiovascular system (especially during the recovery phase) provides valuable information on the cardiac health of the individual [19,20]. For instance, there are conditions when an individual may seem physically fit and healthy, however the self-organization of the cardiovascular system exhibits poor response during the stress test. That could be an alarming condition indicating early symptoms of serious problems. 

This article introduces yet another interpretation of the collapse of complexity of the cardiovascular system during the stress test. The time-delayed time difference pattern, the Shan wavelet coefficient pattern, accompanied by relevant statistical analysis yields a promising technique for the determination of the cardiovascular response during the stress test.

The health indicator introduced in this paper is based on the interplay between the values of $A_{d}$ and $A_{r}$ and the distribution of the coefficients of the Shan wavelet. The importance of the balance between two indicators α and β can be clearly observed even for the small cohort studied in this paper. For example, there have been occasions where a person, despite belonging to the cohort of healthy individuals, has been classified into the warning zone in terms of their cardiovascular health (and vice versa). 

The presented methodology is built on algorithms with open architecture. Similar open-architecture algorithms are also presented in [6,16,17] for diagnostic purposes. The term “open architecture” can be thought of as a machine learning model whose capacity to produce better results increases as more information is integrated into the model. In one of the studies [17], a similar open-architecture scheme is proposed for understanding the complexity of arterial blood pressure regulation in a cohort of individuals with normal blood pressure and those with a history of hypertension. The algorithms used in [17] are based on the matrix architecture known as the Perfect Matrices of Lagrange Differences. Furthermore, the classification and decision support system techniques are built upon three sets of conditions to indicate the cardiovascular performance for each individual.

In our study, we propose a similar open-architecture approach for diagnostic purposes. This unique algorithm is based upon a time-delayed pattern to generate a second-order matrix for evaluation of cardiac intervals (duration of the JT wave and the RR interval). For generating the decision support system, health indicators are thoughtfully proposed, aiding in the visualization of the cardiovascular health status for each individual in the entire cohort. The introduced health indicator gamma is based on two parameters, alpha and beta. It is clearly demonstrated that a single parameter α and β are not sufficient alone to diagnose early indications of problems occurring during stress test. The structure of the algorithm allows convenient extension of the number of parameters representing different aspect of the cardiovascular system dynamics. The introduction of these new parameters would only make the health indicator gamma more specific. Adding new parameters and extending the cohort is a definite objective of future research. As mentioned already, the open architecture of the proposed approach is indeed a unique feature. The larger the cohort, the more reliable the Gaussian approximations leading to the classification of individuals according to the developed performance indicators. However, it is already demonstrated that even the small cohort produces reliable Gaussian approximation according to the Anderson Darling test. 

It is important to note that this study introduces a novel technique to understand the collapse of complexity and the self-organization of the cardiovascular system during the stress test. Due to the novelty of the study theme, it cannot be directly or indirectly compared to other studies in terms of result efficiency because it is one of the efforts to demonstrate that a matrix-based approach and health indicators can be effectively used for analyzing complex signals like ECG.

However, the algorithm and the of approach of this novel work is comparable to the works of Ziaukas et al. [16] and others [17,35] who have also used matrix-based algorithms and approaches to analyze ECG signals.

Despite the modest sample size, the study serves as an effective platform to visualize the effect of the collapse of complexity (during the load) and the self-organization (during the stress test) of the processes during the mobilization of an individual’s cardiovascular system. The proposed technique is not only capable to classify healthy and unhealthy individuals but can also generate early warnings.

### 3.3. Limitations

One of the primary limitations of this study is the relatively small sample size utilized for the analysis. It is well understood that a larger dataset would likely yield more refined outcomes. Another limitation of the study is the absence of female participants in the cohort. Including both male and female participants would allow for a comprehensive investigation of cardiovascular behavior during the stress test, providing a broader perspective on the collapse of complexity and the self-organization processes in both groups. Nonetheless, the aim of this study is to demonstrate that the proposed approach is effective even with a limited dataset, and that incorporating more data would further enhance the efficacy of the algorithm.

### 3.4. Conclusions

The time-delayed pattern approach established in this work provides an unprecedented opportunity to observe the time-shifted relationship between the RR and JT time series. This allows observation of the variations of the collapse of complexity as well as self-organization of the cardiovascular system during the stress test via a distinct perspective. In addition to the delayed pattern approach, the velocity of the adaptation index and the modified velocity of the adaptation index imparted comprehensive details about the cardiovascular mobilization. Finally, the wavelet time frequency analysis approach supplemented the findings by determining the cardiovascular system response during the stress and was shown to be a useful tool in establishing the health biomarker. The results of this analysis can be useful for diagnostic purposes of early symptoms of problems associated to the collapse of complexity and the self-organization of the cardiovascular system during the load and recovery phases of the stress test.

## Figures and Tables

**Table 1:** Experimental protocol for the entire cohort.

Candidate	Day/Month/Year	Local Time (Eastern European Time)	
		Start of Experiment	End of Experiment
H_1_	04/06/2022	09:00	10:00
H_2_	04/06/2022	10:05	11:05
H_3_	04/06/2022	11:05	12:05
Break hour			
H_4_	04/06/2022	13:05	14:05
U_1_	04/06/2022	14:05	15:05
U_2_	04/06/2022	15:05	16:05
U_3_	04/06/2022	16:05	17:05
U_4_	04/06/2022	17:05	18:05

**Table 2:** Formulization of the health indicator α for each individual based upon $A_{r}$ and $A_{d}$ values.

Persons from Cohort	Velocity of Adaptation Index, $A_{d}$	Modified Velocity of Adaptation Index, $A_{r}$	Formulization, $X=\frac{\left(A_{d}-A_{r}\right)}{A_{d}}\times 100\%$	Parameter, $\alpha =\frac{X-min}{max-min}\times 100\%$
$H_{1}$	14.34	12.81	10.66	56.17
$H_{2}$	7.74	6.73	13.04	63.18
$H_{3}$	9.71	7.23	25.54 *	100
$H_{4}$	15.83	17.14	−8.27	0.41
$U_{1}$	4.72	4.38	7.20	45.97
$U_{2}$	9.56	9.75	−1.98	18.93
$U_{3}$	9.9	8.38	15.35	69.98
$U_{4}$	7.72	8.37	−8.41 **	0

The single * and double asteria ** signs indicate the minimum and maximum values.

## Data Availability

The data presented in this study are available on request from the corresponding author.

## Abbreviations

CNS	central nervous system
ABP	arterial blood pressure
HR	heart rate
MET	metabolic equivalent
SV	stroke volume
ABP (sys)	systolic arterial blood pressure
ABP (dis)	diastolic arterial blood pressure
JTa	amplitude of the JT interval
JTd	the duration of the JT interval
ECG	electrocardiogram
AHA	American Heart Association
